# Holographic Photolysis for Multiple Cell Stimulation in Mouse Hippocampal Slices

**DOI:** 10.1371/journal.pone.0009431

**Published:** 2010-02-25

**Authors:** Morad Zahid, Mateo Vélez-Fort, Eirini Papagiakoumou, Cathie Ventalon, María Cecilia Angulo, Valentina Emiliani

**Affiliations:** 1 Wavefront-Engineering Microscopy Group, Neurophysiology and New Microscopies Laboratory, CNRS UMR 8154, INSERM U603, University Paris Descartes, Paris, France; 2 Neuron-Glia Interactions Group, Neurophysiology and New Microscopies Laboratory, CNRS UMR 8154, INSERM U603, University Paris Descartes, Paris, France; The Research Center of Neurobiology-Neurophysiology of Marseille, France

## Abstract

**Background:**

Advanced light microscopy offers sensitive and non-invasive means to image neural activity and to control signaling with photolysable molecules and, recently, light-gated channels. These approaches require precise and yet flexible light excitation patterns. For synchronous stimulation of subsets of cells, they also require large excitation areas with millisecond and micrometric resolution. We have recently developed a new method for such optical control using a phase holographic modulation of optical wave-fronts, which minimizes power loss, enables rapid switching between excitation patterns, and allows a true 3D sculpting of the excitation volumes. In previous studies we have used holographic photololysis to control glutamate uncaging on single neuronal cells. Here, we extend the use of holographic photolysis for the excitation of multiple neurons and of glial cells.

**Methods/Principal Findings:**

The system combines a liquid crystal device for holographic patterned photostimulation, high-resolution optical imaging, the HiLo microscopy, to define the stimulated regions and a conventional Ca^2+^ imaging system to detect neural activity. By means of electrophysiological recordings and calcium imaging in acute hippocampal slices, we show that the use of excitation patterns precisely tailored to the shape of multiple neuronal somata represents a very efficient way for the simultaneous excitation of a group of neurons. In addition, we demonstrate that fast shaped illumination patterns also induce reliable responses in single glial cells.

**Conclusions/Significance:**

We show that the main advantage of holographic illumination is that it allows for an efficient excitation of multiple cells with a spatiotemporal resolution unachievable with other existing approaches. Although this paper focuses on the photoactivation of caged molecules, our approach will surely prove very efficient for other probes, such as light-gated channels, genetically encoded photoactivatable proteins, photoactivatable fluorescent proteins, and voltage-sensitive dyes.

## Introduction

In recent years, the use of advanced optical techniques has been generating a continuously growing interest in the field of neurobiology not only for visualizing neuronal structures and signaling processes, but also for controlling neuronal and glial cell activity. This has been made possible by a rapidly expanding set of photosensitive neurotransmitters that can be precisely controlled by light excitation (photolysis) [1]. In the field of neurobiology, photolysis of caged molecules has been actively used to explore different physiological processes amongst which synaptic receptor activation, neuronal synaptic plasticity and neuronal network functioning [2]. Single- and two-photon uncaging has also been important to demonstrate the Ca^2+^-dependent release of gliotransmitters by astrocytes in acute brain slices [3], [4], [5], [6] and to elucidate the role of this glial cell type in neuro-vascular coupling [7], [8]. In addition to caged neuroactive compounds, genetically encoded light-sensitive proteins [9] and synthetics photoswitches [10] have recently been developed, permitting neuronal activation and inhibition in freely moving animals [11], [12], [13]. In conjunction with spatiotemporally resolved photo-stimulation techniques, these photosensible tools represent the most promising alternative to electrical stimulation, providing ways to control precisely in space and time the activity of specific types of brain cells. These approaches require fast, flexible and precise illumination schemes, permitting a selective activation and imaging of sub-cellular regions or multi-cellular ensembles, with enough power to drive reactions quickly and fast gating.

We recently proposed a novel method to generate flexible and precise light patterning [14]. This method, highly sophisticated but relatively easy to implement, exploits a Liquid Crystal Spatial Light Modulator (LC-SLM). Following the notion of image reconstruction in holography, the principle follows from calculating (with an iterative algorithm) a phase pattern at the rear aperture of the objective that allows reproducing a given target intensity at the objective focal plane, e.g. a fine neuronal structure such as pieces of dendrites [14]. The calculated phase-hologram is addressed to the LC-SLM that is designed to impose the phase modulation onto the input beam wavefront. After propagation through the objective, the beam is focused onto an illumination pattern reproducing straight away the desired template with sufficient light intensity for fast uncaging. This is in stark contrast to digital mirror devices, where a large fraction of laser power is lost because the intensity patterning is created by redirecting unwanted light out of the excitation field [15], [16], [17]. Acousto optical deflectors (AOD), which quickly direct the full power of the laser over the region of interest [18], are limited by the residence and travel time to what can be considered to be ‘simultaneously’ activated on the millisecond biological timescale [19], [20].

We have also shown that for large excitation areas holographic illumination has a significantly higher axial resolution than a Gaussian beam and further optical confinement can be achieved in two-photon (2P) holographic illumination combined with temporal focusing (TF) [21], [22]. In proof-of-principle experiments, we used the 1P holographic method to release caged glutamate in brain slices and showed that shaped excitation on segments of neuronal dendrites and simultaneous multi-spot excitation of different dendrites enables a precise and rapid spatio-temporal control of glutamate receptor activation [14]. Recently it has been proved that a similar scheme for 2P excitation works also for glutamate uncaging in brain slices [23].

Here, we extend the use of holographic photolysis for the excitation of multiple neurons and of glial cells. To implement holographic photoactivation for multiple cells stimulation, we added several additional features to the original holographic microscope. This includes the use of the HiLo microscopy [24], [25] (High frequency/Low frequency sequential acquisition) to improve the contrast and accuracy of primary fluorescent images, and the removal of the zero order spot to enlarge the excitation field. By means of electrophysiological recordings and calcium imaging in acute hippocampal slices, we show that the use of excitation patterns precisely tailored to the shape of multiple neuronal somata represents a very efficient method for the simultaneous excitation of a group of neurons. Indeed, holographic photostimulation easily generates action potentials and Ca^2+^ signals on target neurons with a relatively high spatial resolution and low excitation density. In addition, we demonstrate that fast shaped illumination patterns also induce reliable responses in single glial cells. Taking oligodendrocyte precursors expressing the proteoglycan NG2 (NG2 cells) [26] as a cellular model, we demonstrate that holographic photolysis efficiently elicits glutamatergic currents with fast kinetics and intracellular Ca^2+^ signals in these cells.

## Results

### Modification to the Original Holographic Microscope

**Figure 1:**
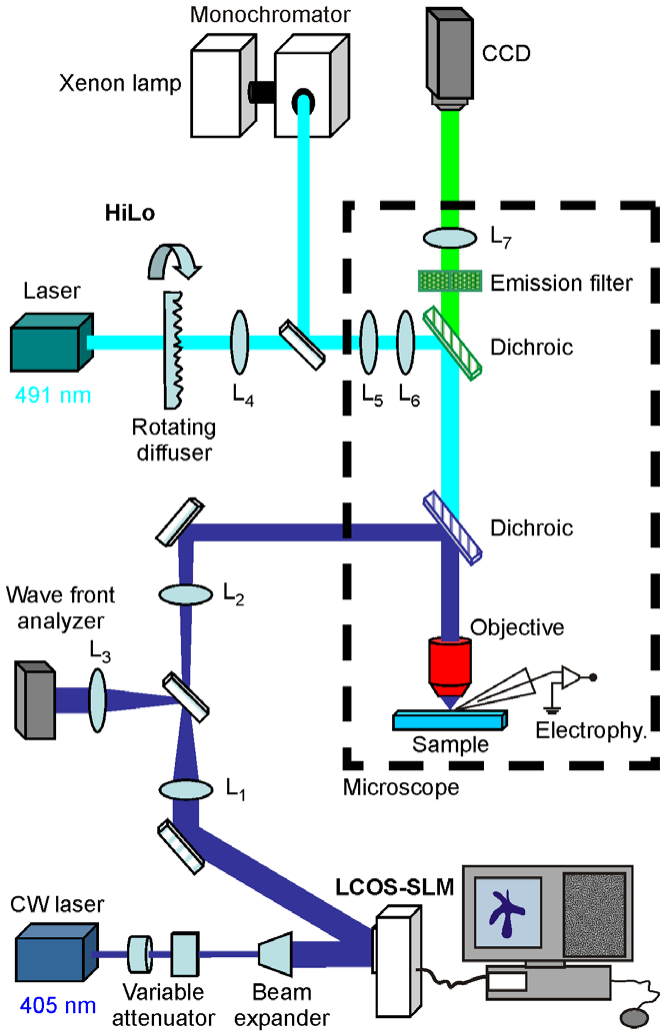
Optical set-up for holographic illumination. Layout of the optical set-up. The focal length of the lenses are f_1_ = 750 mm, f_2_ = 500 mm, f_3_ = 200 mm, f_4_ = 75 mm. See methods for details.

**Figure 2:**
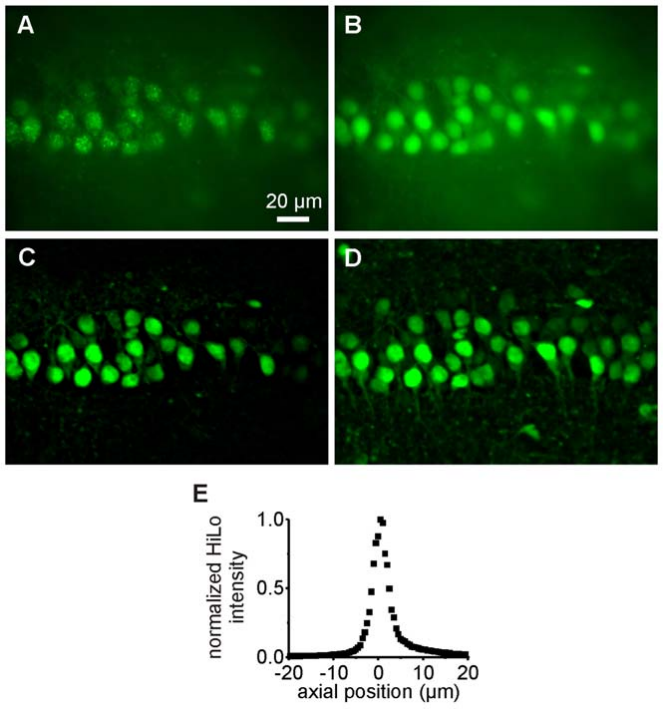
Optical sectioning with HiLo microscopy. **A–C.** Speckle (A) and uniform (B) illumination images used in the calculation of the quasi-confocal HiLo image (C). **D.** z-projection of 12 HiLo sections (including C) separated by Δz = 2 µm. **E.** Measurement of the HiLo microscope axial resolution: integrated signal from a thin fluorescent layer (≈0.3 µm) as a function of defocus z. The measured axial resolution is 4 µm FWHM.

The holographic microscope (Figure 1) is similar to the one described in Ref. [14]. Briefly, it is an epi-fluorescence upright microscope equipped with an additional illumination path for bringing in the laser beam containing the holographic signal. In this path, an input beam from a 405 nm CW laser diode is converted into a holographic beam by the use of a LCOS-SLM, controlled by a custom-designed software (see Methods). Two modifications were made to better implement multi-cell excitation. First, the excitation field was enlarged to ∼80×80 µm^2^. This was accomplished by axially displacing the zero-order spot away from the objective focal plane (by illuminating the LCOS-SLM with a slightly converging beam) while maintaining the +1^st^-order spot at the objective focal plane using a suitable hologram addressed to the LCOS-SLM [27]. Doing so allows spatially filtering the zero order spot with an external blocker with negligible effects on the propagation of the +1^st^-order beam. As a result, we could dispose of a full field of view of ∼80×80 µm^2^ (see Methods and Figure S1). A second modification was to improve the contrast and accuracy of the primary fluorescent image based on which the 3D pattern for holographic excitation signal will be generated (Figure 2). This is achieved by outfitting the microscope with a recently developed microscopy technique called “HiLo” microscopy [24] (see Methods). Briefly, this method relies on the acquisition of two fluorescence images and the use of a post-processing algorithm to reconstruct a quasi-confocal sectioned image. The first image, in our case of a neuron population in an Oregon Green BAPTA-1AM (OGB) loaded hippocampal slice, is obtained with structured illumination and more precisely here (as in [24]) with speckle illumination (Figure 2A). Evaluating the local contrast on this image yields a sectioned image of low resolution. To obtain the missing high frequencies, we acquire a second fluorescent image using a uniform illumination (Figure 2B). By applying a high-pass filter to this latter image, we extract its high-frequency components which inherently exhibit sectioning. Combining these two post-processed images therefore produces a quasi-confocal image containing all frequency components. Figure 2 shows a comparison between wide-field epi-fluorescence (Figure 2B) and HiLo microscopy (Figure 2C) images. A clear improvement in respect to a conventional widefield image appears evident: the background has been significantly reduced and the contour of the cells appears more distinctive, thus facilitating the generation of the holographic illumination pattern for uncaging experiments. Figure 2D represents a z-projection of 12 sections (including Figure 2C) separated by Δz = 2 µm. With the parameters used in this paper (see Methods), the axial resolution is 4 µm at the Full Width at Half Maximum (FWHM; Figure 2E).

### Holographic Illumination to Stimulate Multiple Cells: Lateral and Axial Distribution

**Figure 3:**
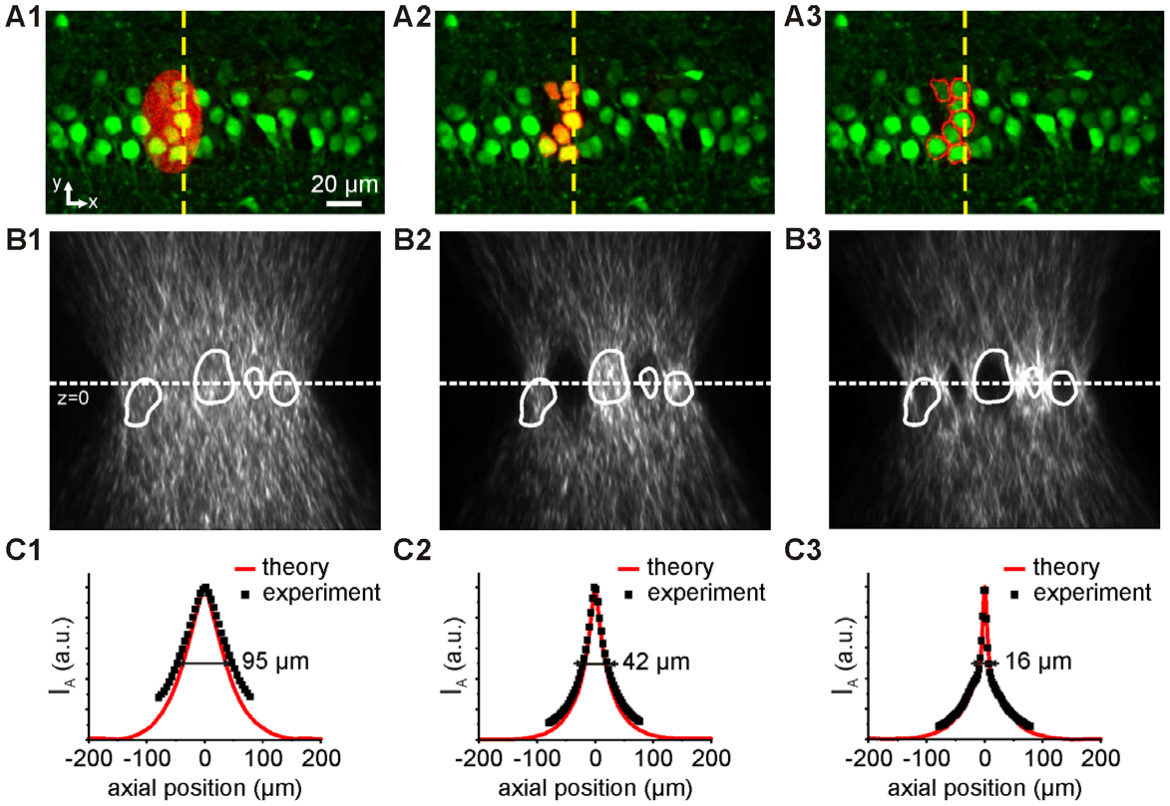
Holographic multiple cell photostimulation. **A.** Overlay of a fluorescence image recorded with HiLo microscopy showing OGB-loaded CA1 neurons of a hippocampal slice with three different excitation pattern configurations for uncaging: elliptic, shaped (cell somata) and anti-shaped (extracellular space) patterns. The superimposed red images of the excitation spots were obtained by exciting a thin layer of fluorescein. **B.** Measured y-z intensity cross-sections of the excitation beam along the yellow lines shown in A. The contour lines of OGB-loaded cells imaged with HiLo microscopy are superimposed (white lines). **C.** Distributions of calculated (red) and measured (black) axial intensities for the spots in A. The calculation was based on Angular Spectrum of Planar Wave Approximation algorithm [48].

Figures 3 A1–A3 show a HiLo fluorescence image of OGB-loaded neurons of the CA1 pyramidal cell layer from an hippocampal slice superimposed on three different holographic patterns (red) designed to excite 7 cells simultaneously: an ellipse covering the whole area encompassing the cells (A1), a pattern shaped to selectively cover the 7 cell somata (shaped pattern; A2), and a pattern shaped to excite the extracellular space immediately adjacent to the 7 cells (anti-shaped pattern; A3).

The axial propagation for each of the three holographic patterns is examined by using a double microscope where the upper objective is scanned through a thin fluorescent layer and where the imaging objective is fixed and focused on the sample (see Methods). Figures 3 B1–B3 show the corresponding y–z intensity cross sections taken along the yellow lines of Figures 3 A1–A3. To estimate the axial excitation achieved with those patterns in respect to the cells, we draw on Figures 3 B1–B3 the contour lines of the cells, imaged with HiLo microscopy.

To estimate the axial resolution corresponding to each illumination configuration, we measured, for each axial plane of the 3D intensity stack, the intensity, I_A_, integrated over a region of interest equal to excitation shape at z = 0 (Figures 3 C1–C3; integrating the intensity results in averaging the inhomogeneities due to the speckles). We then define the range of focus, *b*, as the FWHM of the corresponding intensity profiles I_A_(z). From the experimental curves, we found for the elliptic, shaped and anti-shaped patterns a value of *b* = 95±0.5 µm, 42±0.5 µm, and 16±0.5 µm respectively, in good agreement with the values of 81 µm, 37 µm, and 17 µm obtained with the theoretical curves (red-solid line; for details on the calculation see Methods). All the curves have an asymptotic behavior proportional to ∼1/z^2^.

This study shows that the use of a shaped pattern adjusted to selectively excite several cell somata allows reaching an axial resolution of ∼40 µm with an extremely precise lateral distribution of excitation light. Such a pattern also allows concentrating the activation region to the full somata membrane with reduced ‘unwanted’ photoactivation of other regions. A further gain in axial resolution and excitation density can be reached by selectively redirecting light on the extra-cellular space in proximity of the target cells (anti-shaped pattern). This solution should also reduce photodamage considering that most of the excitation light is redirected outside of the cell body. Moreover it represents a logical choice for photolysis since the extracellular space is where the caged compound is located in most experimental situations.

### Holographic Photolysis to Stimulate a Group of Neurons

**Figure 4:**
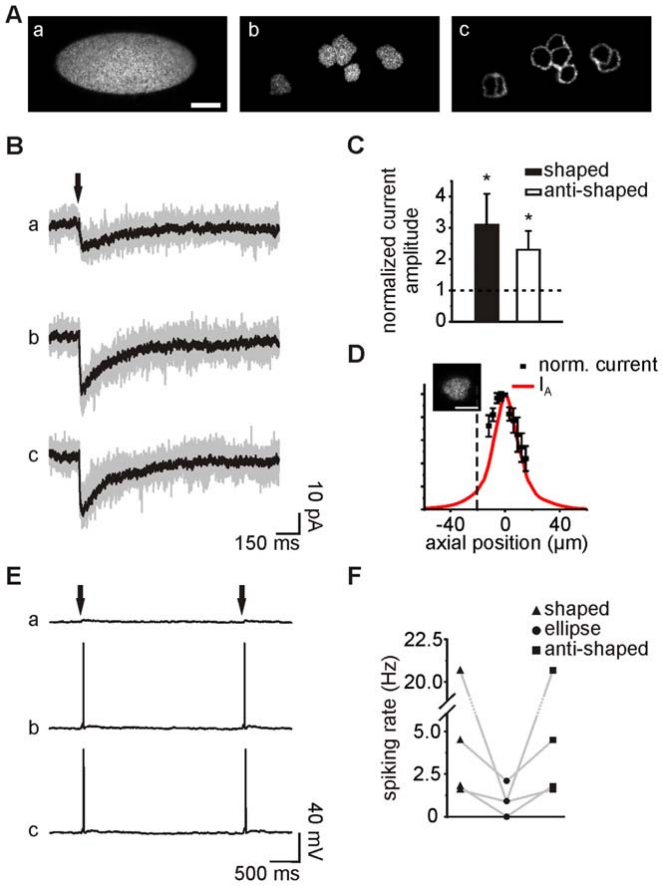
Electrophysiological recordings upon holographic photostimulation of multiple neurons. **A.** Images of three pattern configurations obtained by exciting a thin layer of fluorescein: ellipse (a), shaped (b) and anti-shaped (c) patterns. Scale bar: 20 µm. **B.** Photolysis-evoked currents elicited in a recorded CA1 neuron held at −60 mV by uncaging MNI-glutamate in a group of target cells with the three patterns in (A), using an energy of 3.5 µJ at the sample plane. Individual sweeps (gray) and averaged currents (black) are shown (excitation densities: 0.9 nJ/µm^2^, 3.6 nJ/µm^2^ and 10 nJ/µm^2^ for the elliptic, shaped and anti-shaped patterns, respectively). **C.** Histogram of current amplitude increases obtained with shaped and anti-shaped patterns normalized by the currents obtained with an ellipse. **D.** Mean normalized amplitudes of photolysis-evoked currents obtained in five recorded neurons held at −60 mV and excited with a shaped pattern, while changing the focal plane of the objective with steps of 3 µm. The top of the slice is indicated by the black dashed line. The theoretical axial distribution of light (I_A_) for a shaped pattern is superimposed. Scale bar for the inset: 10 µm. **E.** Photolysis-evoked responses recorded in the same cell as in (B) in current-clamp mode. **F.** Comparison of spiking rates obtained with the three pattern configurations in four distinct cells.

To compare the effect of the different excitation configurations represented in Figure 3A, we performed several experiments in which we used similar excitation patterns (a large ellipse or circle, a shaped pattern and an anti-shaped pattern; Figures 4Aa–c; 800 µs laser pulses) to uncage MNI-glutamate while monitoring the effects by whole-cell patch-clamp recordings or Ca^2+^ imaging.

In a first set of experiments, we used the same excitation energy for the three excitation patterns which, depending on the number of target cells in each experiment (from 5 to 11 target neurons per slice), corresponded to excitation densities of 0.6–2.2 nJ/µm^2^, 2.8–12.1 nJ/µm^2^ and 5.4–33.3 nJ/µm^2^ for the elliptic, shaped and anti-shaped patterns, respectively. In all cases, the values were largely below the photodamage threshold of 500 nJ/µm^2^ as estimated in reference [28]. Initially, a selected pyramidal cell from the target neurons was recorded with a patch pipette at a holding potential of −60 mV in voltage-clamp mode. Figures 4Ba-c illustrates the inward currents elicited by the three different illumination patterns in the recorded neuron. The three configurations induced highly reproducible inward currents that, in all tested neurons, were characterized by coefficients of variation of 0.35±0.12, 0.17±0.06 and 0.19±0.05 for the elliptic, shaped and anti-shaped spots, respectively (n = 5 cells in 5 different slices). Yet, consistently with the use of higher excitation densities, the normalized current amplitudes were 2 and 3-fold larger for the anti-shaped and shaped patterns than for the elliptic pattern (Figure 4C; n = 5 cells in 5 different slices; P<0.05).

To demonstrate the sectioning capabilities of our method, we measured the photolysis-evoked currents in recorded neurons excited with a holographic pattern shaped to cover the whole soma (Figure 4D, inset), while axially displacing the illumination pattern with respect to recorded neurons (by moving the objective with steps of 3 µm). By plotting the axial distribution of the mean normalized currents for five cells as a function of the objective focal plane position, we found a good agreement with the theoretical axial propagation expected for the corresponding holographic beam (Figure 4D; n = 5 cells in 5 different slices; for details on the calculation see Materials and Methods).

To further test the efficiency of shaped and anti-shaped patterns to stimulate simultaneously multiple target neurons in brain slices, we compare the capacity of different pattern configurations to elicit action potentials. In accordance with the results described above, the lower excitation densities achieved with the elliptic illumination pattern elicited none or few action potentials in cells recorded in current-clamp mode. Conversely, shaped and anti-shaped illumination patterns always triggered more action potentials in the same cells (Figures 4E and 4F; n = 4 cells in 4 different slices).

**Figure 5:**
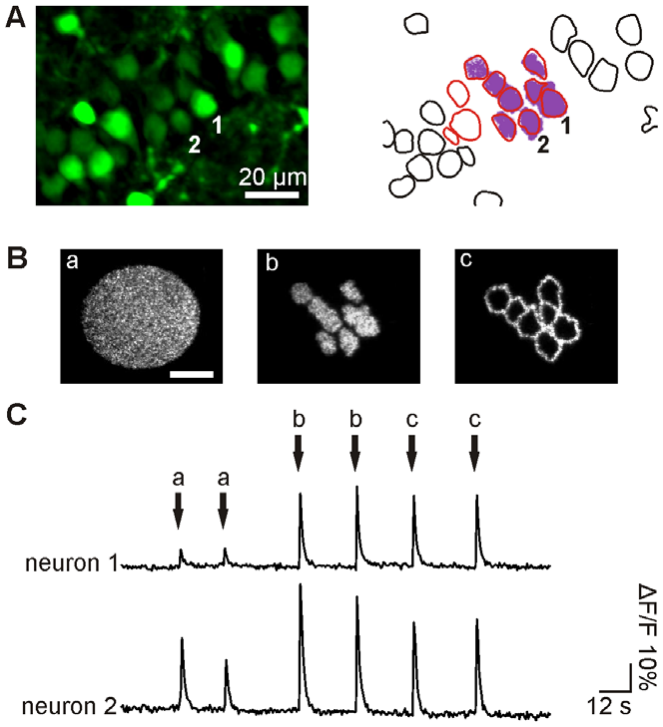
Ca^2+^ imaging upon holographic photostimulation of multiple neurons. **A**. (left) Fluorescence images obtained with HiLo microscopy showing CA1 neurons of a hippocampal slice. (right) Scheme of responding (red) and non-responding (black) cells to photostimulation with a shaped pattern (blue). **B**. Images of the three patterns used to photostimulate target neurons in CA1 with MNI-glutamate using the same energy of 3.6 µJ, corresponding to 1.7 nJ/µm^2^, 6.2 nJ/µm^2^ and 18.1 nJ/µm^2^ for the elliptic, shaped and anti-shaped patterns, respectively. **C**. Variation of intracellular Ca^2+^ concentration of two different target neurons during photostimulation with the three different pattern configurations.

To assess simultaneous activation of the group of photostimulated neurons, we examined changes in intracellular Ca^2+^ concentration by following the fluorescence of OGB (Figure 5A, left). In these experiments, we used the same energy for all excitation patterns, corresponding to excitation densities of 1.1–1.7 nJ/µm^2^, 4.4–9.1 nJ/µm^2^ and 10.9–19.7 nJ/µm^2^ for the elliptic, shaped and anti-shaped patterns, respectively (from 6 to 10 target neurons per slice). In these conditions, elliptic, shaped and anti-shaped illumination patterns elicited intracellular Ca^2+^ increases in 84%, 94% and 94% of target neurons, respectively (Figure 5B; n = 32 cells in 4 slices). In three out of four slices, few non-target neurons also responded with intracellular Ca^2+^ elevations to the stimulation (Figure 5A, right), but these responding cells were always located within a distance of 20 µm from stimulation patterns (average distance from spots: 17.5±5.6 µm). Considering the sharp lateral distribution of excitation light with the holographic system [14], these responses were probably caused by the excitation of neuronal dendrites passing close to the excitation spots and the diffusion of glutamate in the slice. Consistent with the use of higher excitation densities, shaped and anti-shaped patterns induced 2-fold larger increases of intracellular Ca^2+^ signals than the elliptic pattern (Figure 5C; P<0.01). Moreover, the Ca^2+^ responses to two consecutive photostimulations did not change significantly in all tested cells (Figure 5C; P>0.05).

Taken together, these results demonstrate that the generation of holographic patterns precisely tailored to the shape of multiple neuronal somata represents a very efficient way for a quick excitation of a group of neurons. This configuration has the advantage over the use of large spots to increase, for a given excitation power, the effective excitation density available for the experiment. Indeed, we have shown that low-energy, multiple shaped and anti-shaped patterns generate effective photostimulation as revealed by larger inward currents, action potential discharges and Ca^2+^ signal increases in target neurons. To reach an excitation density comparable to that of the shaped pattern, the ellipse excitation area needs to be reduced at least by a factor of 2–3, thus limiting the number of excitable cells. Moreover, we demonstrated that the use of a holographic phase – compared with an almost flat phase, typical of a Gaussian beam - allows reaching a good axial resolution, even for a large excitation area encompassing the whole cell soma.

The use of anti-shaped patterns allows for a further improvement of the axial resolution and excitation density (Figure 3). However, neuronal responses remained similar to those elicited with shaped patterns at energies inducing non-saturating responses (Figures 4, 5 and S2). This probably implies that, with anti-shaped patterns, the higher concentration of effective light and thus of uncaged glutamate at the focal plane is compensated by the fact that the top and bottom membranes of some cells are less excited than with shaped patterns (those membranes being located up and below the focal plane).

### Holographic Photolysis to Stimulate NG2 Cells

In addition to the application of photosensitive tools for the understanding of neuronal function, photolysis of caged compounds has also been used to activate glial cells of the brain. Here, we tested whether holographic photolysis constitutes a suitable tool for fast stimulation of non-neuronal cells, taking as a cellular model NG2 cells (oligodendrocyte precursors). This glial cell type, characterized by a small cell soma and very thin processes extending from the cell body, expresses ionotropic glutamate receptors and receives synaptic inputs from neurons [29]. It has been speculated that the fast activation of these receptors may be involved in different physiological roles such as regulation of proliferation and differentiation [26]. Holographic photolysis could be an interesting tool to activate rapidly and selectively ionotropic receptors of NG2 cells in brain slices. Since NG2 cells express Ca^2+^-permeable and Ca^2+^-impermeable AMPA receptors in the hippocampus [29], [30], [31], we tested the conditions to detect glutamatergic currents and Ca^2+^ signals induced by a holographic photoexcitation of the cell soma.

**Figure 6:**
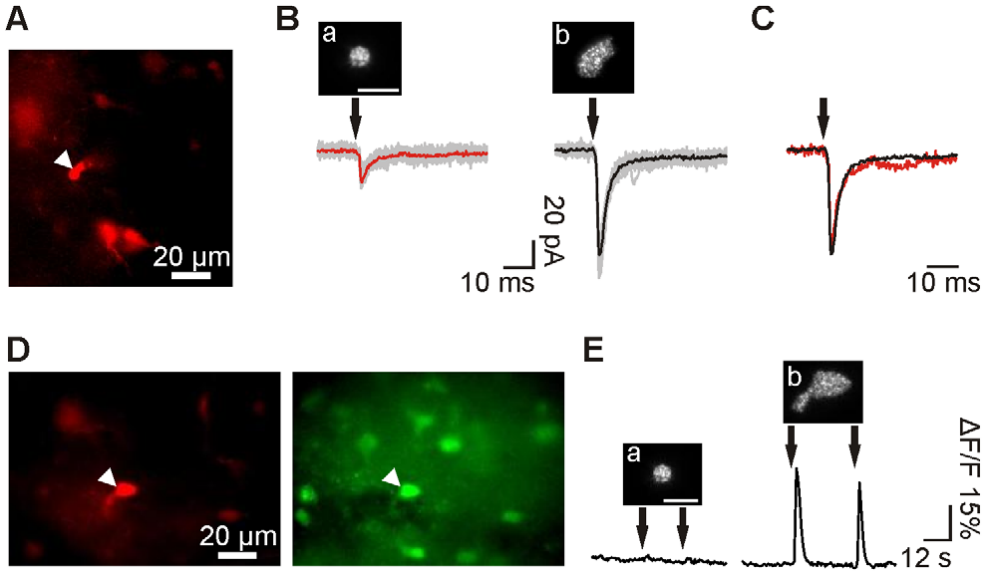
Holographic photostimulation of NG2 cells. **A.** Red fluorescence image of a target DsRed^+^ NG2 cell (arrowhead) in a hippocampal slice of NG2-DsRed transgenic mice. **B.** Photolysis-evoked currents elicited in the same cell held at −70 mV upon illumination with a 5 µm spot (a) and shaped pattern (b), using the same power density (120 nJ/µm^2^). Individual sweeps (gray) and averaged currents (red and black) are shown. **C.** Normalized photolysis-evoked currents shown in (B). Note the similarity of the kinetics. **D.** Fluorescence images of a target DsRed^+^ NG2 cell in a OGB-loaded hippocampal slice of NG2-DsRed transgenic mice (arrowheads). **E.** Variation of intracellular Ca^2+^ concentration during photostimulation with a 5 µm spot (a) and shaped pattern (b), using the same power density (100 nJ/µm^2^). Scale bars for insets: 10 µm.

NG2 cells expressing the fluorescence protein DsRed were recorded from NG2-DsRed transgenic mice (Figure 6A). Single DsRed^+^ NG2 cells held at −70 mV were stimulated by uncaging MNI-glutamate at the level of the soma. In these experiments, we compared the responses induced in NG2 cells by two patterns of different sizes but same power density: 5 µm circular spot vs shaped pattern. Similarly to what we showed for neurons, holographic photolysis evoked highly reproducible inward currents in NG2 cells, characterized by coefficients of variation of 0.13±0.04 and 0.22±0.01 for the 5 µm spot and shaped pattern, respectively (Figure 6B). Higher current amplitudes were observed by increasing the illumination area to cover the whole cell soma (Figure 6Bb), indicating that the number of activated AMPA receptors increased with the pattern size (−10.93±3.13 pA vs −43.9±9.89 pA for the 5 µm spot and the shaped pattern, respectively, n = 5; P<0.01; excitation density comprised between 60 nJ/µm^2^ and 120 nJ/µm^2^). Despite these changes in amplitude, the rise time (t_10–90%_) and the weighted decay time (τ) were unaffected by the change in illumination area, indicating that glutamate clearance was similar in both cases (Figure 6C; t_10–90%_: 0.85±0.15 ms vs 0.76±0.13 ms and τ: 31±19 ms vs 42±13 ms for the 5 µm spot and the shaped pattern, respectively; P>0.05).

We could have increased the area of the circular spots to cover the same cell region than with the shaped patterns. For example, the recorded cell in Figure 6D could has been covered with a spot of 20 µm. However, this would correspond to an inevitable deterioration of the axial resolution (40 µm for a large circular spot vs 20 µm for the shaped pattern). Thus, although large circular spots covers the same number of receptors than the shaped pattern, the restricted diffusion volume of glutamate for a shaped pattern permits increasing axial resolution and accelerating photolysis-evoked currents [14].

It has been previously shown that long-lasting applications (30 s) of 500 µM glutamate through puff pipettes induce intracellular Ca^2+^ concentration increases in NG2 cells [31]. We evaluated whether fast activation of AMPA receptors with short pulses in the millisecond range was also able to induce intracellular Ca^2+^ responses on DsRed^+^ NG2 cells loaded with OGB. For these experiments, we used 5 µm spots and shaped patterns of similar power densities (Figures 6D and 6E; excitation density comprised between 80 nJ/µm^2^ and 120 nJ/µm^2^). Again the possibility of adapting the excitation pattern was determinant to optimize the signal. While illumination with a 5 µm spot elicited small responses in 2 of 7 cells (ΔF/F of 6.6±5.0), shaped illumination patterns induced larger intracellular Ca^2+^ elevations in 4 out of 7 DsRed^+^ NG2 cells (Figure 6E; ΔF/F of 16.4±6.0). To discard the possibility that these responses correspond to random spontaneous activity of the cells, we applied two successive stimulations separated by 25 s. We observed reproducible Ca^2+^ responses with ΔF/F of 16.4±6 and 15.5±5 for the first and second pulse, respectively (n = 4; P>0.05). Our results demonstrated that the possibility to increase the excitation area of holographic patterns offers a suited tool to perform photolysis of NG2 cells in brain slices, inducing fast receptor activation and intracellular Ca^2+^ increases.

## Discussion

An efficient way to quickly stimulate several cells is to deliver the excitation light on a large area. This has been achieved with lens-based systems and laser- or lamp-based illumination methods [9], [32], [33], [34], [35], [36], [37]. However, these approaches are limited to the use of circular patterns and require modifying the optical set up for any adjustment of the excitation spot size. Moreover, they are based on the use of Gaussian beams, so that an increase of the excitation spot size gives rise to a rapid loss in axial resolution (quadratic dependence). For example, generating an excitation area that can cover multiple neurons, i.e. a circle of at least 50 µm in diameter, extends the axial resolution to more than 1 mm, so that multiple cell layers are inevitably photoexcited. Conversely, we have shown that shaped holographic illumination allows enlarging the excitation area with a reduced cost in axial resolution. This result can be explained by considering that, unlike the under-filling Gaussian beam method, spatial shaping of the excitation light for a holographic beam results from the interference of multiple beams with a wide range of incident angles, thus permitting to take advantage of the large numerical aperture of the objective. This effect is more pronounced for illumination patterns with high spatial frequency components (i.e. a wider angle range), as for example in the case of the anti-shaped spot [14].

We have compared three types of holographic illumination patterns to stimulate simultaneously a group of neurons: large circular or elliptic patterns, patterns shaped to target multiple cell somata and anti-shaped patterns adjusted to fill their proximal extracellular space. We have found that the most efficient method to specifically target a group of cells is the second solution, i.e. to generate shaped illumination patterns that selectively excite the whole cell soma. This approach allows working at excitation densities well below the photodamage threshold and reducing the generation of by-products [28]. Moreover, it reduces the axial resolution to around two pyramidal cell layers. This axial resolution is particularly suitable for photolysis experiments requiring multi-cell excitation, as it insures that all neurons located near the focal plane are equally stimulated, even if they lay in slightly different axial planes. This situation is difficult to achieve with higher resolution patterns, such anti-shaped patterns or strongly focalized spots.

Alternatively, patterned excitation can be reached by using digital micromirror devices (DMDs) with amplitude modulation [15], [16], [17]. However, this approach has a very low efficiency, as the excitation patterns are created by redirecting light away from the excitation field. As an example, the excitation patterns of Figures 4Aa-c generated with a DMD would give an excitation efficiency, η =  pattern intensity/total intensity, of 10%, 3%, 0.1%, respectively while with the holographic patterns we measured a diffraction efficiency of 47%, 44% and 41%. Moreover, the amplitude modulation of the excitation beam achieved with DMDs maintains a flat optical wavefront. As a consequence, the axial propagation resembles that of a Gaussian beam, yielding to a rapid loss in axial resolution for increased lateral extension of the excitation spot (Figure S3).

Quick excitation of multiple cells can also be achieved by fast scanning a single laser beam [18], [38], [39], [40]. Although this approach is well adapted to mimic dendritic integration of multiple synaptic events, it has two major limitations for stimulating multiple cells. First, the small volume of a diffraction limited spot (∼3–8 µm^3^) limits the effective number of released molecules [41], [42]. We have actually shown, in agreement with previous findings, that this configuration was unable to elicit responses in neurons for excitation power densities below the damage threshold (Figure S4). One possible solution to increase the fraction of excited membrane is to scan the excitation beam through several uncaging locations over the soma [18], [20], [40], [41]. However, the limited temporal resolution intrinsic of this method drastically restricts the maximum number of excitable cells. For example, the area of Figure 4Ab, instantaneously excited with a holographic beam, would require an excitation time of ∼6 s for a spot of 1 µm and pulse length of 800 µs.

Finally, we have shown that the implementation of HiLo microscopy allows generating fluorescence images with reduced background, improved contrast and almost confocal optical sectioning. In this way, the input pattern to the IFTA algorithm calculating the holographic phase profile can be quickly generated using gray-level threshold detection, once a defined region of interest in the image is selected.

In conclusion, we have presented the use of holographic illumination for efficient excitation of neurons and glial cells. We have shown that, in comparison to other existing approaches, the main advantage of holographic illumination is that it allows working at low excitation power densities and relatively high axial resolution. Another advantage of this technique is that excitation pattern areas can be easily tuned to optimize the amplitude of the evoked signals, under conditions of constant power density. One of the aims of multiple neuron photostimulation in brain slices has been to trigger action potentials in presynaptic neurons in order to characterize neuronal circuits [40], [43], [44]. Although one-photon excitation has been used in this type of studies [43], [44], the poor axial resolution of this approach did not allow to determine precisely the neurons under control. Two-photon excitation has solved this problem as photoexcitation is highly confined around the focal plane, allowing the excitation of individual cells [40]. However, fast activation of multiple neurons in a submillisecond range is not achievable with this technique because scanning of multiple sites over the cell soma is required [40]. To date, holographic illumination is the most suited method allowing for a fast and efficient excitation of a group of cells, with a good axial resolution. The versatility of the holographic tool could be of particular interest to study neuronal circuits, but also the bidirectional communication between neurons and glia in the brain. Indeed, the stimulation patterns can be adjusted according to the target cell type. Althought in the present report we have limited the validation of holographic illumination to photolysis of MNI-glutamate, our approach will surely prove very efficient for other probes, such as intracellular cages, genetically encoded photoactivatable proteins, photoactivatable fluorescent proteins, and voltage-sensitive dyes.

## Materials and Methods

### Ethics Statement

Animal procedures were approved by “Direction Départementale des Services Vétérinaires” (DDSV) of “Ministère de l'agriculture et de la pêche” and were in compliance with the European Union institutional guidelines for the care and use of laboratory animals (Council directive 86/609EEC). Mice were housed two adults per cage and maintained with *ad libitum* access to food under light (12 hours light/dark cycle; lights on at 7:00 AM) and temperature (22±2°C) controlled conditions.

### Holographic Light Pattern Generation

The holographic microscope (Figure 1) consists of an epi-fluorescence upright microscope (Olympus BX50WI) equipped for electrophysiological recordings. As a source for uncaging experiments, we use a 405 nm diode CW-laser (CUBE 405-100, Coherent). The output beam is expanded (11X) to match the input window of a LCOS-SLM (X10468-01, Hamamatsu), which operates in reflection mode. The device is controlled by a custom-designed software already described in [14]. Briefly, given a target intensity distribution at the focal plane of the microscope objective, the software calculates the corresponding phase-hologram and addresses the pattern to the LCOS-SLM. The program uses the Iterative Fourier Transform Algorithm (IFTA) [45] to adjust the size and shape of illumination volumes. The algorithm is implemented on graphic card processors GeForce 9400 GT (NVIDIA) yielding a calculation time of ∼30 iterations/s.

A wavefront analyzer (SID4-028 Phasics S.A.) positioned at a plane conjugated with the LCOS-SLM is used to measure the optical wavefront distortions induced by the LCOS-SLM and all the elements located in the optical path between the laser source and the lens L_1_ (Figure 1). A correction phase mask, Φ_C_(x,y), is generated (software kindly provided by Phasics S.A.) and addressed to the LCOS-SLM to compensate the wavefront distortions and to restore a flat phase at the SID4-CCD plane. The correction phase mask, Φ_C_(x,y), is then added to the phase profile, Φ(x,y), calculated for generating a given target intensity, so that the total phase modulation at the LCOS-SLM is Φ_SLM_(x,y)  = (Φ_C_(x,y)+ Φ(x,y))mod2π.

A 4f telescope (L_1_; f_1_ = 750 mm, L_2_; f_2_ = 500 mm) is used to image the LCOS-SLM plane to the rear aperture of the objective (Olympus, LUMPLFL40XW/IR; NA = 0.8) via a dichroic mirror (DM; Chroma Technology 425DCXR) mounted into a dual port tube (Olympus, U-DP) located between the objective and the fluorescence filters wheel of the microscope.

One limitation of digital holography using liquid crystal SLMs (LC-SLMs) is that only a limited portion of the incident light is sent into the target pattern (the 1^st^ order), while the rest is distributed into the “ghost” image, diffracted symmetrically with respect to the 1^st^ order, and into an un-diffracted component which forms a central spot (zero order) in the excitation field [27]. One possibility to remove these unwanted components from the excitation field is to perform a spatial filtering. To this end, a phase grating is introduced in the phase mask, Φ_SLM_(x,y), that is Φ_SLM_(x,y)  =  (Φ_spot_(x,y) + Φ_grating_(x,y) + Φ_C_(x,y)) mod2π. In this way the 1^st^ order and its ghost image are symmetrically displaced from the zero order spot and a diaphragm placed at the intermediate Fourier plane allows selecting only the 1^st^ order for propagation into the objective focal plane. However, this approach reduces the excitation field to ∼¼ of the available excitation area [14], [46]. Although, with the new generation of LC-SLM devices (LCOS) the contribution from the ghost images has been strongly reduced, eliminating the zero order spot without hindering the use of the central part of the excitation field is still desirable.

To this end, we use here a different method, originally developed to increase the excitation field of multiple-trap optical tweezers [27]. This approach consists in introducing a wavefront curvature Φ_lens_(x,y) on the beam incident on the SLM by modifying the alignment of the first beam expander. As a result, the zero-order and 1^st^-order spots, originally focused at the objective focal plane, z_0_, come to focus at a distance z_0_+Δz. A phase term Φ-_lens_(x,y) is then added in the phase mask addressed on the SLM so as to compensate the induced beam curvature Φ_lens_(x,y). This brings back the 1^st^ order spot into to the original position z_0_ and leaves the zero order spot at z_0_+Δz (Figure S1). A small beam blocker placed at the conjugate plane of z_0_+Δz allows removing the zero-order component from the excitation field with negligible effects on the propagation of the 1^st^ order beam. We found that with Δz = +25 µm the zero-order component can be suppressed with negligible effect on the intensity of the 1^st^ order beam even when the latter is placed at a central position (Figure S1). It is worth noting that for devices which deviate a significant portion of light also into the ghost image (e.g. optically addressed LC-SLMs), a similar scheme will axially move this component symmetrically with respect to the zero order (so in this case the ghost image would be generated at z_0_+2Δz, i.e. 50 µm away from the focal plane), so that its effect at the focal plane is negligible. As a result, excitation spots can be located within all the available excitation field available, i.e. 
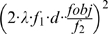
, where d is the spatial frequency corresponding to the maximum deflection angle from LCOS-SLM and f_obj_ the objective focal length. In our experimental condition, this corresponds to a region of ∼80×80 µm^2^ or 100×100 µm^2^ if we limit the area to a diffraction efficiency ≥60% (d = 15 lp/mm) or ≥40% (d = 20 lp/mm), respectively.

### Fluorescence Imaging

High-resolution fluorescence imaging of brain slices has been performed using HiLo microscopy, a new optical sectioning microscopy technique developed by the group of J. Mertz [24]. This technique requires the acquisition of two fluorescence images: one with uniform illumination, and one with structured illumination. In our case (as in Ref. [24]) the structured illumination image is obtained by projecting a speckle pattern in the sample, which is easily produced by introducing a diffuser plate (light shaping diffuser 10°, from kit 20DKIT-C1, Newport) in the laser beam path (laser Cobolt Calypso, ⌊  = 491 nm), in a plane conjugated with the microscope objective back-aperture. The uniform illumination image is obtained by randomizing the speckle patterns, i.e. by rapidly rotating the diffuser with a stepper motor (Nanotec, ST5709S1208-B) while acquiring the image. The sectioned image is computed with Matlab (2008a).

With HiLo microscopy, a compromise can be made *a posteriori* between the resolution and the signal-to-noise ratio. Here, the sectioned image is intended to be used by the custom-designed software described earlier, in order to design the holographic patterns. Since this software first applies an automatic threshold to the image, we decided to give priority to a high signal-to-noise ratio at the expense of the resolution. In that goal, we first apply a low-pass filter to the uniform illumination image in order to reduce the shot-noise (this decreases the lateral resolution to about 1 µm FWHM). In addition, we apply a wavelet filter to the speckle illumination image, in order to reduce the out-of-focus background (as described in [47]) and the shot-noise. We then apply the algorithm described in [24] to those two pre-filtered images to compute the sectioned image. The cut-off frequency used to merge the low and high-frequency components is chosen to k_c_≈0.1k_low_, where k_low_ is the frequency of the low-pass filter applied to the uniform illumination image. With those parameters, we measure an axial resolution of 4 µm FWHM (see Figure 2E). This axial resolution was evaluated by measuring the HiLo signal from a thin layer of Rhodamine 6G spin-coated on a coverslip (thickness ≈0.3 µm) as a function of the axial distance z between the fluorescent layer and the focal plane of the objective.

The HiLo technique was easily implemented on the microscope Olympus BX51 by adding a dual port tube (Olympus, U-DP) right before the microscope illumination path, thus allowing the user to choose between speckled laser light (for HiLo microscopy) and a monochromator light (for calcium imaging, see below). We found that using the full microscope illumination path to generate structured illumination patterns is perfectly compatible with speckle illumination, while grid patterns – which can also be used in HiLo microscopy [25] – proved to be more sensitive to aberrations (in particular, distortion) introduced by the microscope lens L_5_.

For Ca^2+^ imaging, excitation light (488 nm; 10 nm bandwidth) was provided by a 75-W Xenon lamp passing through a monochromator (Optoscan, Cairn Research) and fluorescence images were collected with a CCD cooled (–30°C) 12-bit CCD camera (CoolSNAP HQ2, Roper Scientific). Emission wavelengths were obtained by using a HQ535/50M filter (Chroma Technology). Metamorph software was used to acquire and store images for off-line analysis in Matlab (7.0). Exposure time was 50 ms and images were collected at 2 Hz. The background fluorescence was subtracted and Ca^2+^ responses were expressed as relative changes in fluorescence (ΔF/F). Traces showing ΔF/F larger than 2% were considered as responses.

Analysis of holographic beam propagation around the objective focal plane has been done with a double microscope already described in Ref. [14], [22]. In this system the upper objective (LUMPLFL40xW/IR, Olympus) is used to generate the holographic excitation volume. Different sections of the holographic beam are obtained by varying, with a piezo objective positioner (NV40/ICL E; Piezosystem Jena), the position of the objective with respect to a thin fluorescent layer (thickness  = 0.3 µm). The lower objective (UPLSAPO60xW) is used to collect the fluorescence from the fluorescein sample and is kept at a fixed position.The collected fluorescence from the sample is imaged by a collection lens (150 mm focal length) to a CCD camera (CoolSNAP HQ2, Roper Scientific). To reject the excitation light, an emission filter (HQ 535/50M, Chroma Technology) was placed in front of the CCD camera. Optical sectioning and image acquisition have been performed using Metamorph software (version 7.5; Molecular Devices).

To compare the experimental axial propagation with the theory, we have implemented in the software the possibility of determining, for each phase-hologram, Φ_SLM_(x,y), the tri-dimensional intensity profile of the corresponding beam around the objective focal plane. Briefly, given an input phase-hologram Φ_SLM_(x,y) and a Gaussian intensity distribution I_Gauss_(x,y), at the SLM plane, we calculate the beam irradiance around the objective focal plane, after the diffracted electromagnetic field at the SLM plane 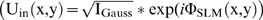 is propagated through the optical path comprising the two lenses L1, L2 and the objective lens. Calculation was done by using the thin element approximation (TEA) in the angular spectrum approach of plane waves (ASPW) [48]. To determine the intensity distribution I_Gauss_(x,y) at the SLM plane, we expanded the intensity beam profile measured at the exit of the laser (FWHM  = 0.7 mm) by a factor 11 (corresponding to the expansion of the beam expander before the SLM).

### Slice Preparation and Loading

Heterozygous NG2-DsRed BAC transgenic mice [49] were crossed with C57BL/6 mice to generate transgenic and wild-type littermates that were used to perform acute hippocampal slices (300 µm) from postnatal day 4 (PN4) to PN11 as we previously described [50]. After preparation, slices were loaded for 1 h at 33°C with the cell-permeant calcium indicator OGB (11 µM) in a recording solution containing (in mM): 126 NaCl, 2.5 KCl, 1.25 NaH_2_PO_4_, 26 NaHCO_3_, 20 glucose, 5 pyruvate, 1 CaCl_2_ and 2 MgCl_2_ (95% O_2_, 5% CO_2_). Then, the slices were transferred into a recording chamber perfused with the same solution at 2 mL/min. A recycling perfusion system was used to minimize recording solution volumes (5 mL) and allow the continuous perfusion of the caged MNI-glutamate (1 mM).

### Electrophysiology

Neurons of CA1 pyramidal layer and NG2 cells of CA1 stratum radiatum were visualized using IR-DIC video microscopy. NG2 cells were identified by detecting the fluorescence of DsRed. Excitation light for DsRed (560 nm; 585 nm bandwidth) was provided by a 75-W xenon lamp coupled with a monochromator (Optoscan, Cairn Research) and emission wavelengths were obtained by using a HQ640/40M filter (Chroma technology). Patch-clamp recordings were performed in whole-cell configuration at 32°C in voltage-clamp and current-clamp modes. Neurons were recorded with an intracellular solution containing (in mM): 130 KCl, 5 EGTA, 0.5 CaCl_2_, 2 MgCl_2_, 10 HEPES, 2 Na_2_ATP, 0.2 Na-GTP, 10 Na_2_-phosphocreatine (pH≈7.3). The intracellular solution for NG2 cells contained (in mM): 130 CsCl, 10 4AP, 5 TEA-Cl, instead of 130 KCl, to minimize potassium conductances. Recordings were made without series resistance compensation. Series resistances were monitored during recordings and cells showing a change of more than 30% were discarded.

Whole-cell recordings were obtained using Multiclamp 700B, filtered at 2–4 kHz and digitized at 10 kHz. Digitized data were analyzed off-line using pClamp 10.1 software (Axon Instruments). Neurons and NG2 cells were recorded at a holding potential of −60 mV and −70 mV, respectively. Amplitude variations of photolysis-evoked currents were estimated by calculating the coefficient of variation of the responses. The mean amplitudes, decay and rise times (t_10–90%_) of currents were calculated by averaging 5 to 15 traces. The spiking rate for each cell was obtained from 20 individual traces. Data were expressed as mean ± s.e.m. The statistical significance was determined using a Student *t* test.

## Supporting Information

Figure S1 Enlargement of the excitation field. A. Phase hologram (Φspot) calculated by the IFTA to generate, for an incident collimated beam, a centered circular spot (1st order beam) of 15 µm diameter focalized at the objective focal plane; phase grating (Φgrating) introduced to laterally shift the target spot from the zero order; correction phase mask (Φc) used to anneal the optical aberrations. Final phase mask (ΦSLM) sent to the SLM resulting from the sum of all these phase-components, that is ΦSLM(x,y)  =  (Φspot(x,y) + Φgrating(x,y) + ΦC(x,y))mod2π. B. Fluorescence image of the spot generated at the objective focal plane (z = 0) with ΦSLM, showing the centered zero order spot and the laterally displaced 1st order spot C. Lens effect (Φlens) used for illumination with a divergent beam to compensate the wavefront curvature of beam. When this term is added to ΦSLM(x,y): (Φ'SLM(x,y)  =  (Φlens(x,y) + ΦSLM(x,y))mod2π) the zero order and the spot are axially separated. The 1st order is focalized at the objective focal plane (z = 0 µm), and the zero order spot at +25 µm from the objective focal plane. D. Fluorescence images of the zero order at z = +25 µm and of the 1st order spot at z = 0 µm. Please note that the phase grating (Φgrating) is still present in Φ'SLM(x,y), resulting in a lateral displacement of the 1st order beam in addition to the axial displacement. We chose this configuration to show more clearly the effect of the defocused first order spot at the focal plane (light halo). The zero order can be blocked at the z = +25 µm conjugate plane. E. Phase hologram without the phase grating (Φ' 'SLM(x,y)  =  Φspot(x,y) + Φlens(x,y) + ΦC(x,y))), which places the 1st order spot at the center of the focal plane F. Fluorescence images of the 15 µm spot placed either at the center (left) or at the side (right) of the excitation field, generated by the phase holograms Φ' 'SLM and Φ'SLM, respectively. Scale bars for insets: 10 µm.
(9.27 MB TIF)

Figure S2 Ca2+ imaging responses induced by different energies. Averaged intracellular Ca2+ signals of target neurons elicited by shaped and anti-shaped patterns as a function of excitation energy, for the slice shown in Fig. 5 (n = 8 cells). This plot shows that Ca2+ responses are not saturated by the energy of 3.6 µJ (arrow) used for Ca2+ imaging experiments.
(1.29 MB TIF)

Figure S3 Holographic spot vs. spot generated with DMDs. A. Simulated intensity profile (left up), phase distribution (left down) and axial propagation for a 10 µm spot generated with Digital Micromirror Devices (DMD). B. Simulated intensity profile (left up), phase hologram (left down) and axial propagation for a 10 µm spot generated with digital holography. C. Integrated intensity over the area of the spots shown in (A) and (B) as a function of the axial position. Note the difference in the axial confinement in the two cases.
(4.57 MB TIF)

Figure S4 Comparison of neuronal responses elicited by a strongly focalized spot and a shaped pattern. A. Lateral intensity distribution of a strongly focalized spot; the fit with a Gaussian function gives a lateral FWHM of ∼1 µm. B. Axial intensity distributions IA for the strongly focalized spot shown in (A) (black) and for the shaped pattern (blue) used in (C). C. Patch-clamp recordings of a neuron held at −60 mV upon illumination with (a) a focalized spot and (b) a shaped pattern, using the same power density (40 nJ/µm^2^). Images of the two spots are shown in insets. Scale bar: 10 µm. D. Histogram of the current amplitude obtained with both spots. Note the absence of response with the strongly focalized spot (n = 4). These experiments demonstrate that a pattern shaped to cover the whole cell soma allows working at low excitation power. We needed to raise the excitation density to 400 nJ/µm2 to generate detectable photolysis-evoked currents with strongly focalized spots (10.0 pA, in one out of three cells; not shown), i.e. to a value close to the photodamage threshold (500 nJ/µm^2^) [27]. In contrast, illumination with a shaped spot covering the whole cell soma at power densities of 40–60 nJ/µm^2^ evoked large inward currents in all cases.
(4.34 MB TIF)
